# Indinavir and atazanavir; comparison of predicted property by chemoinformatics technique and implication on renal problem in HIV infected patients

**Published:** 2016-01-21

**Authors:** Beuy Joob, Viroj Wiwanitkit

**Affiliations:** ^1^Sanitation 1 Medical Academic Center, Bangkok, Thailand; ^2^Visiting Professor, Hainan Medical University, Haikou, China ; Visiting Professor, Faculty of Medicine, University of Nis, Serbia; adjunct professor, Joseph Ayobabalola University, Nigeria; Honorary Professor, Dr DY Patil Medical University, India

**Keywords:** Atazanavir, Indinavir, Renal, Human immunodeficiency virus

**Implication for health policy/practice/research/medical education:**

Anti-HIV medication is the standard treatment for the HIV infected patient. An important concern of anti-HIV drug use is the adverse effects. The kidney problems due to anti-HIV is widely mentioned. Indinavir and atazanavir are the two drugs with many reports on renal toxicity. Here, the authors use the standard chemoinformatics technique for predicting properties of both drug and compare the derived result. Based on this study, atazanavir seems to be more problematic than indinavir considering the induction of renal problem.

## Introduction

HIV infection becomes the present problematic infectious disease worldwide. The pathogenic HIV virus will destroy immune system of the patients and result in several clinical problems. Anti-HIV medication is the standard treatment for the HIV infected patient. An important concern of anti-HIV drugs use is the possible adverse effects (1). The kidney problems due to anti-HIV is widely mentioned. Indinavir and atazanavir are the two drugs with many reports on renal toxicity (2). Focusing on the pathogenesis, the crystal formation is believed to be the important pathological process. Here, the authors use the standard chemoinformatics technique for predicting properties of both drug and compare the derived result.

## Materials and Methods

This work is a chemoinformatics study. The standard technique namely is “General Unrestricted Structure*-*Activity Relationships – GUSAR” used. The online prediction tool is available at http://cactus.nci.nih.gov/. The technique is used in previously published article (3). In this work, the two studied drugs are indinavir and atazanavir. The three main properties relating to crystal formation, melting point, solubility and viscosity are assessed.

## Results

Based on this study, the predicted melting points of indinavir and atazanavir are equal to 186.8 and 205.34ºC, respectively. The solubility of indinavir and atazanavir are equal to -3.208 and -3.719 log^10^ (mol/l), respectively. The viscosity of indinavir and atazanavir are equal to 2.675 and 3.302 log^10^ (CP), respectively.

## Discussion

The main pathogenesis of renal problem due to antiretroviral drug is the crystal formation (2). Based on this study, the melting point of indinavir and atazanavir are comparative studied. Here, it can see that both substances have melting point higher than the room temperature confirmed that both drug is dissolvable. This can support that the drug can be dissolved after oral intake and further absorb by human body for further action. Focusing on the solubility, which is the main factor determining the formation of crystal. It can be seen that atazanavir has poorer solubility which implies higher risk to become crystal. In addition, the viscosity of atazanavir also has higher viscosity (viscosity is the main determinant for needle shaper crystal formation; see more details from http://www.thaiceramicsociety.com/). This implies the change of higher risk of needle shape crystal formation than indinavir.

Based on this work, the authors use the standard chemoinformatics technique for predicting properties of both drug and compare the derived result. Based on this study, atazanavir seems to be more problematic than indinavir considering the induction of renal problem. In fact, both drugs can cause the renal problem and the problem is not uncommon (4,5). However, the case of atazanavir seems to cause more serious problem including to renal failure. Nevertheless, the authors assess three important properties of studied drugs, melting point, solubility and viscosity. In real life, there are also other factors to be observed.

## Authors’ contribution

VW and BJ wrote the manuscript equally.

## Conflicts of interest

The author declared no competing interests.

## Ethical considerations

Ethical issues (including plagiarism, data fabrication, double publication) have been completely observed by authors.

## Funding/Support

None.
